# Evaluation of a Novel Renewable Hepatic Cell Model for Prediction of Clinical CYP3A4 Induction Using a Correlation-Based Relative Induction Score Approach[Fn FN3]

**DOI:** 10.1124/dmd.116.072124

**Published:** 2017-02

**Authors:** Rongjun Zuo, Feng Li, Sweta Parikh, Li Cao, Kirsten L. Cooper, Yulong Hong, Jin Liu, Ronald A. Faris, Daochuan Li, Hongbing Wang

**Affiliations:** Corning Life Sciences, Bedford, Massachusetts (R.Z., F.L., S.P., L.C., K.L.C.); Corning, Science and Technology, Corning, New York (Y.H., J.L., R.A.F.); and University of Maryland, School of Pharmacy, Baltimore, Maryland (D.L., H.W.)

## Abstract

Metabolism enzyme induction-mediated drug-drug interactions need to be carefully characterized in vitro for drug candidates to predict in vivo safety risk and therapeutic efficiency. Currently, both the Food and Drug Administration and European Medicines Agency recommend using primary human hepatocytes as the gold standard in vitro test system for studying the induction potential of candidate drugs on cytochrome P450 (CYP), CYP3A4, CYP1A2, and CYP2B6. However, primary human hepatocytes are known to bear inherent limitations such as limited supply and large lot-to-lot variations, which result in an experimental burden to qualify new lots. To overcome these shortcomings, a renewable source of human hepatocytes (i.e., Corning HepatoCells) was developed from primary human hepatocytes and was evaluated for in vitro CYP3A4 induction using methods well established by the pharmaceutical industry. HepatoCells have shown mature hepatocyte-like morphology and demonstrated primary hepatocyte-like response to prototypical inducers of all three CYP enzymes with excellent consistency. Importantly, HepatoCells retain a phenobarbital-responsive nuclear translocation of human constitutive androstane receptor from the cytoplasm, characteristic to primary hepatocytes. To validate HepatoCells as a useful tool to predict potential clinical relevant CYP3A4 induction, we tested three different lots of HepatoCells with a group of clinical strong, moderate/weak CYP3A4 inducers, and noninducers. A relative induction score calibration curve-based approach was used for prediction. HepatoCells showed accurate prediction comparable to primary human hepatocytes. Together, these results demonstrate that Corning HepatoCells is a reliable in vitro model for drug-drug interaction studies during the early phase of drug testing.

## Introduction

Cytochrome P450 (CYP) enzymes are a major elimination pathway through which many drugs are metabolized. The expression levels of several CYPs involved in drug metabolism can be induced by drugs and other xenobiotics through nuclear receptor-mediated pathways, for example, the pregnane X receptor (PXR), the constitutive androstane receptor (CAR), and the aryl hydrocarbon receptor (Bjornsson et al., 2003; Park et al., 2004; Mueller et al., 2006; Mohutsky et al., 2010). CYP induction by xenobiotics could affect the pharmacokinetics of coadministered drugs, causing potential therapeutic failure by increasing the clearance of victim drugs if the coadministered drug is a substrate of the affected enzymes, or leading to hepatotoxicity by increasing accumulation of reactive drug metabolites, or resulting in altered pharmacokinetic profiles of coadministered drugs if the victim drugs are pro-drugs (Hebert et al., 1992; Hewitt et al., 2007). In addition, CYP induction could also cause nonstationary pharmacokinetics if the victim drug itself is an inducer, namely autoinduction (Löscher and Schmidt, 2006). Because CYP induction could pose a significant risk to patients, induction-mediated drug-drug interaction (DDI) needs to be carefully evaluated to determine and/or predict their safety risk.

Primary human hepatocytes are considered the gold standard in vitro model system for drug metabolism and induction studies. Currently, both the Food and Drug Administration (FDA) (http://www.fda.gov/downloads/Drugs/GuidanceComplianceRegulatoryInformation/Guidances/ucm292362.pdf) and European Medicines Agency (EMA) (http://www.ema.europa.eu/docs/en_GB/document_library/Scientific_guideline/2012/07/WC500129606.pdf) recommend using primary human hepatocytes as an in vitro test system for studying CYP induction. However, primary human hepatocytes are well recognized as having large donor-to-donor variations and limited supply of high-quality donor tissues (Shimada et al., 1994; Roymans et al., 2005). It is therefore required to screen multiple lots of primary human hepatocytes for better prediction accuracy, which is a lengthy and costly procedure for researchers. To overcome the limitations inherent in primary human hepatocytes, we have created a renewable source of human hepatocytes, HepatoCells, as an alternative to primary human hepatocytes. These cells are manufactured and cryopreserved under defined conditions to ensure lot-to-lot consistency. In this study, we describe the characterization of HepatoCells for in vitro CYP induction assays and the evaluation of using HepatoCells as an in vitro tool to screen test compounds for potential clinical induction liability.

As the most important drug-metabolizing enzyme, CYP3A4 metabolizes about half of the drugs on the market; therefore, it is critical to study induction involving CYP3A4. Currently, several models have been proposed to predict clinical CYP3A4 induction using in vitro concentration response data from primary human hepatocytes. These models, ranging from simple to complex, include correlation-based models such as C_max_/EC_50_ and relative induction score (RIS), basic static model R_3_, and mechanistic models such as net effect model and physiologically-based pharmacokinetic model (Fahmi and Ripp, 2010; Fahmi et al., 2012; Einolf et al., 2014). Although all these models show reasonable prediction accuracy, we chose the RIS-based correlation approach in the present study for its relative simplicity, sufficient accuracy, and its incorporation in the EMA guidance.

## Materials and Methods

### 

#### Materials and Reagents.

Corning HepatoCells (catalogue 354881) were directly derived from primary human hepatocytes (9-year-old Caucasian female donor). Briefly, the simian virus 40 large T antigen was introduced to the parental cells to make immortal clones that were then screened and selected for CYP induction functionality. Selected high-function clones were expanded to make a working cell bank. Working bank cells were then expanded to passage 33 or 34. To induce differentiation to a mature hepatocyte phenotype, prior to cryopreservation, the immortalizing gene was removed. HepatoCells were then cryopreserved similarly as primary hepatocytes, using cell culture medium supplemented with dimethylsulfoxide (DMSO) and serum. Similar to primary hepatocytes, HepatoCells are stored in the vapor phase of liquid nitrogen. Primary human hepatocytes used in this study were obtained from Corning Gentest hepatocyte inventory with donor livers obtained from reliable organ procurement organizations with informed donor consent. Unless otherwise specified, all assays with HepatoCells were performed using Corning Culture Medium for HepatoCells (catalogue 354882) available from Corning Life Sciences (Bedford, MA). Similar to culture medium for primary human hepatocytes, Corning Culture Medium for HepatoCells contains glucocorticoid, insulin, transferrin, and selenium. Corning BioCoat Collagen I–coated plate, Corning CellGro Penicillin-Streptomycin 100× Solution, Corning Matrigel, fetal bovine serum, and Hank’s balanced salt solution with Ca^2+^ and Mg^2+^ (1× Hanks’ balanced salt solution buffer) were products from Corning Life Sciences (Tewksbury, MA). All the chemicals for induction assays were purchased from Sigma-Aldrich (St. Louis, MO). RNeasy 96kit and DNase I kit (Qiagen, Valencia, CA) were used for RNA isolation. Q-PCR master mix; high-capacity reverse-transcription kit; and TaqMan q-PCR primer sets for CYP1A2, 2B6, and 3A4 (assay ID: Hs00430021_m1 for CYP3A4 primer, Hs03044634_m1 for CYP2B6, and Hs00167927_m1 for CYP1A2) were purchased from Life Technology (Carlsbad, CA).

#### Genotyping.

Frozen cell pellets were prepared and shipped to SeqWright Genomic Services (Houston, TX) for genotyping analysis using Sanger sequencing method.

#### HepatoCells Culture.

Cryopreserved HepatoCells were thawed quickly in a 37°C water bath and transferred to Corning Culture Medium for HepatoCells supplemented with 10% fetal bovine serum and Pen/Strep (plating medium). After the cryo-freezing media was removed by centrifugation at 150*g* for 10 minutes, the cell pellet was resuspended in plating media and cell count was performed with trypan blue. Cells were then seeded in a Corning BioCoat Collagen I–coated plate (500,000 cells/well in 24-well plate or 80,000 cells/well in 96-well plate), and plates were incubated in a 37°C incubator with 5% CO_2_. Four hours after seeding, plating medium was removed, and then matrigel solution prepared in cold Corning Culture Medium for HepatoCells at a final concentration of 0.25 mg/mL was added to the monolayer culture at a volume of 0.5 mL/well in 24-well plates or 0.1 mL/well in 96-well plates. Cells were then returned to the incubator for overnight culture.

#### Compound Treatment of CYP Induction.

Overnight culture of HepatoCells was treated with prototypical inducers for CYP3A4, CYP1A2, and CYP2B6 (10 *µ*M rifampicin, 50 *µ*M omeprazole, and 1 mM phenobarbital, respectively) or solvent vehicle control (0.1% DMSO) freshly made daily in serum-free culture medium. After three consecutive 24-hour treatments, cells were washed once with fresh culture medium, and probe substrates for CYP3A4, CYP1A2, and CYP2B6 (200 *µ*M testosterone, 100 *µ*M phenacetin, 250 *µ*M bupropion, respectively) were then added into the culture at 100 *μ*L/well for a 1-h incubation at 37°C to assess enzyme activity. At the end of substrate incubation with the cells, assay was stopped by removing 80 *μ*L/well of enzyme assay supernatant and mixing with 20 *μ*L/well cold stop solution containing heavy labeled internal standard (e.g., 5 *μ*M 6*β*-hydroxytestosterone-[D_7_] in acetonitrile with 0.1% formic acid for CYP3A4, 10 *μ*M acetamidophenol-^13^C_2_^15^N in acetonitrile with 0.1% formic acid for CYP1A2, and 0.1 *μ*M hydroxybupropion-[D_6_] in acetonitrile with 0.1% formic acid for CYP2B6). The samples were then centrifuged at 4000 rpm for 20 minutes, and supernatants were analyzed by liquid chromatography–tandem mass spectrometry for metabolite formation (6*β*-hydroxytestosterone, hydroxybupropion, and acetaminophen). Cryopreserved primary human hepatocytes were cultured and treated similarly as HepatoCells, and induction of CYP3A4, 1A2, and 2B6 was similarly assessed. Note, due to the limited availability of the original donor cells for HepatoCells, we were not able to include the induction assessment of the donor cells and compare it with that of HepatoCells.

To evaluate the applicability of using HepatoCells as an in vitro tool to predict clinical CYP3A4 inducers, we selected 18 compounds that are known clinical strong inducers, moderate or weak inducers, and noninducers, based on their potency in decreasing area under curve (AUC) of coadministered victim drugs in clinical studies (Zhang et al., 2014). Stock solutions of test compounds were prepared by dissolving each compound in DMSO and serially diluting the solutions in DMSO. Final working solutions were freshly prepared daily by diluting the 1000× stock solutions in culture medium. Three lots of HepatoCells culture were treated with eight concentrations of test compounds. Both enzymatic activity (testosterone 6*β* hydroxylase activity) and mRNA expression were measured as endpoints using LC-MS/MS and real-time reverse-transcription polymerase chain reaction, respectively.

#### mRNA Preparation and Analysis.

After the enzyme assay, cells were washed once with fresh culture medium. mRNA was isolated using a Qiagen RNeasy 96 kit. mRNA transcript level was determined using Applied Biosystems two-step protocol on a 7300 real-time polymerase chain reaction system.

#### Detection of Bile Canalicular Efflux Transporter Multidrug-Resistant Protein 2.

On day 1, HepatoCells were plated on collagen-coated dishes. Four to six hours after seeding, cell monolayer was overlaid with Matrigel solution at 0.25 mg/mL (as described above). Daily medium change was performed from day 2 to day 4 using fresh Corning Culture Medium for HepatoCells. On day 5, the sandwich cultures were incubated with carboxy-dichlorofluorescein diacetate (CDFDA), a multidrug-resistant protein 2 (MRP2) substrate that is metabolized by cytosolic esterases. The fluorescent CDFDA metabolite accumulated in bile canalicular lumens was visualized using fluorescence microscopy.

#### Data Analysis and Curve Fitting.

Both enzyme activity and mRNA transcript level of CYP3A4, 2B6, and 1A2 were measured in triplicate wells. Fold induction measured by enzyme activity was determined by normalizing enzyme activity in the presence of different concentrations of test compounds to enzyme activity in the presence of corresponding solvent vehicle control (0.1% DMSO in culture medium). Fold induction measured by P450 mRNA transcript level was determined using the calculation of 2^−ΔΔCT^ (Zhang et al., 2014). Induction response data points are accepted for curve fitting only when two or three replicates show coefficient of variance less than 40%. Data points that could not meet the criteria are excluded from curve fitting. To show a real induction response change relative to the solvent vehicle control, fold increase, which is defined as fold induction minus 1 (Cheng et al., 2016), is used for curve fitting.

To determine the maximum response (E_max_) and EC_50_, CYP3A4 fold increase was plotted against different concentrations of test compounds to generate a concentration-dependent induction response curve, which was fitted to a sigmoidal Hill 4 parameter equation (Kanebratt and Andersson 2008; Zhang et al., 2014) using SigmaPlot (Systat Software, San Jose, CA), as described: y = E_min_ + [E_max_ − E_min_]/[1 + (EC_50_/x)^b], where y is the induction response, E_min_ is background, E_max_ is the maximum induction response, EC_50_ is the drug concentration achieving 50% of E_max_, x is the drug concentration, and b is the slope of the curve. Only curve fitting with correlation coefficient (R^2^) > 0.85 is accepted. At high concentrations for some compounds, if toxicity or insolubility becomes obvious, such data are excluded from curve fitting. Following the same approach as described by Zhang et al. (2014) and Fahmi and Ripp (2010), for data sets that show no apparent plateau, the observed maximum fold increase is accepted as E_max_ to avoid extrapolating too much above the experimental values and EC_50_ is calculated accordingly from the fitting curve. Also, induction response has to be concentration dependent with observed maximum response greater than 1.4-fold in order for the data set to be used for obtaining EC_50_ and E_max_.

The induction parameter RIS was calculated using unbound C_max_ from the literature (Zhang et al., 2014) and equation described below: RIS = (E_max_ × C_max,ub_)/(EC_50_ + C_max,ub_). A calibration curve was generated by plotting the induction paramter RIS against in vivo data (i.e., observed percentage of midazolam AUC change) for each test compound and fitted to a Hill 3 parameter function using SigmaPlot with the following equation: f = a × x^b/(c^b + x^b), where f is the predicted AUC change, a is the maximum AUC change, b is the slope of the curve, c is the value of induction parameter RIS achieving 50% of AUC change, and x is the RIS value. The resulting fitting equation was used to calculate predicted in vivo AUC change for each compound. Prediction accuracy and prediction bias were then determined by comparing predicted AUC change with observed AUC change, using the 2 metrics root mean square error (RMSE) and geometric mean fold error (GMFE) reported previously (Einolf et al., 2014). Prediction accuracy using HepatoCells was also compared with prediction accuracy using primary human hepatocytes.

## Results

### 

#### HepatoCells Genotype and Morphology.

To characterize the genetic compositions of HepatoCells, we performed detailed genotyping analysis for important CYP enzymes with known polymorphisms, such as CYP2D6, 2C9, and 2C19. HepatoCells exhibit wild-type genotype for all tested alleles of CYP2D6 (*3, *4, *5, *6, *7, *8, *9, *10), CYP2C9 (*2 and *3), and CYP2C19 (*2 and *3), except for CYP2D6*2, where HepatoCells carry a *2*2 allele that is considered to exhibit normal activity. Overall, HepatoCells genotyping results suggest that HepatoCells are representative of a Caucasian population (refer to donor description in *Materials and Methods*). Genotyping of important hepatic transporters such as OAP1B1 (SLCO1B1), OATP1B3 (SLCO1B3), and MRP2 (ABCC2) is ongoing and will be reported separately.

**Fig. 1.**
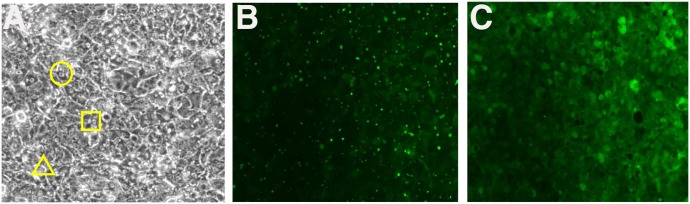
Morphology of HepatoCells. (A) Phase-contrast picture of HepatoCells sandwich culture. The circle indicates polygonal cell shape, the square indicates double nucleation, and the triangle indicates bile canaliculus. (B) Fluorescence image of day 5 HepatoCells sandwich culture in the presence of MRP2 fluorescent substrate CDFDA. (C) Fluorescence image of day 5 HepatoCells sandwich culture in the presence of both MRP2 fluorescent substrate CDFDA and MRP2 inhibitor MK571.

At 24 hours postplating on collagen I Biocoat tissue culture plates, HepatoCells formed a confluent monolayer with the majority of the cells showing mature hepatocyte morphology (Fig. 1A) indicated by distinct polygonal cell shape with clear cell borders, single or multiple round nuclei with prominent nucleoli, and moderate to low nucleus/cytoplasm ratio. Staining of a 4-day culture of HepatoCells with the fluorescent MRP2 substrate CDFDA showed visible bile canaliculus structures, a characteristic of primary hepatocytes in a sandwich culture (Fig. 1B). Pretreatment with the MRP2 inhibitor MK571 inhibited the specific CDFDA staining of bile canaliculi (Fig. 1C). These results suggest that, in addition to similar morphology to primary human hepatocytes, the functional efflux transporter MRP2 is expressed and localized at the apical surface of HepatoCells, consistent with features of mature hepatocytes.

#### CYP3A4, 1A2, and 2B6 Induction Response.

**Fig. 2.**
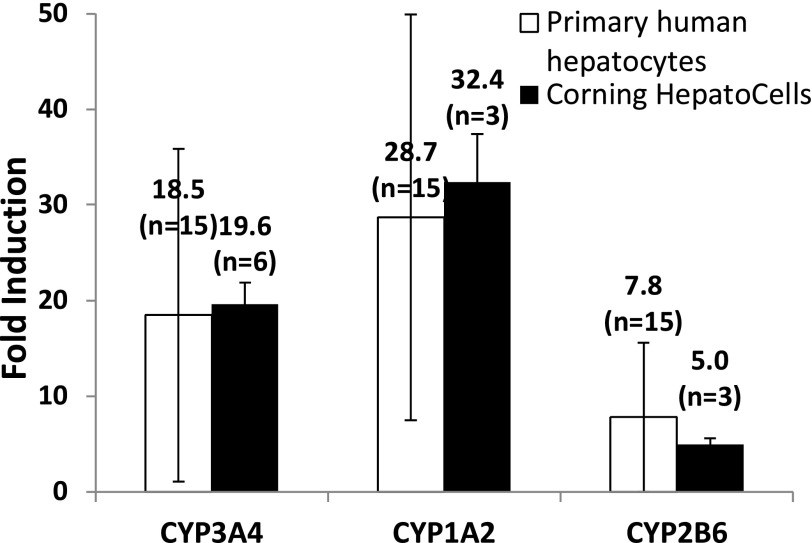
Induction response of CYP3A4, CYP1A2, and CYP2B6 in HepatoCells upon treatment with respective control inducers, 10 *μ*M rifampicin, 50 *μ*M omeprazole, and 1 mM phenobarbital. Enzyme activity in the presence of positive control inducers is normalized to enzyme activity in the presence of solvent vehicle control (0.1% DMSO) to give fold induction. Induction data of primary hepatocytes were generated similarly using cryopreserved human hepatocytes from Corning Gentest inventory. Data shown are mean values of multiple lots. Number of lots used is indicated in parentheses.

To evaluate whether HepatoCells are a useful screening tool for identifying potential CYP inducers, we first examined induction responses of the three important enzymes, CYP3A4, 1A2, and 2B6, in HepatoCells following the industry standard (Sinz et al., 2008; Chu et al., 2009) and FDA-recommended in vitro method (http://www.fda.gov/downloads/Drugs/GuidanceComplianceRegulatoryInformation/Guidances/ucm292362.pdf). Induction response measured by enzyme activity in HepatoCells was compared with primary hepatocytes (Fig. 2). On average, three to six lots of HepatoCells tested showed average fold induction of 20, 32, and 5 for CYP3A4, 1A2, and 2B6, respectively. The average fold induction values obtained with HepatoCells were comparable to the average fold induction obtained from 15 lots of primary human hepatocytes. As expected, different lots of primary human hepatocytes showed large variations in induction responses of all three enzymes, for example, 94% CV for CYP3A4 induction, 74% CV for CYP1A2 induction, and 100% CV for CYP2B6 induction. In contrast, fold induction for individually manufactured HepatoCells lots (3–6 lots) showed much smaller variation, for example, 12% CV for CYP3A4 induction, 15% CV for CYP1A2 induction, and 12% CV for CYP2B6 induction (Table 1).

**TABLE 1:** Lot-to-lot variation (%CV) of HepatoCells versus primary human hepatocytes in induction response The *n* in parentheses indicates number of lots used in the study.

Parameters	Lot-to-Lot Variation (%CV)	
	HepatoCells	Primary Human Hepatocytes
CYP3A fourfold induction	12% (*n* = 6)	94% (*n* = 15)
CYP1A twofold induction	15% (*n* = 3)	74% (*n* = 15)
CYP2B sixfold induction	12% (*n* = 3)	100% (*n* = 15)

#### CAR Nuclear Translocation in HepatoCells.

**Fig. 3.**
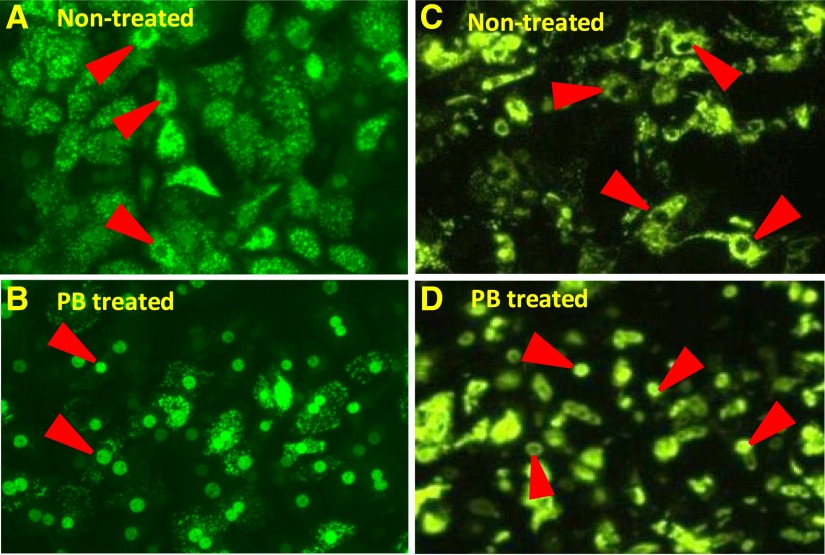
Nuclear translocation of hCAR in HepatoCells and primary hepatocytes upon exposure to phenobarbital. (A) and (B) are primary human hepatocytes before and after phenobarbital treatment, respectively. (C) and (D) are HepatoCells before and after phenobarbital treatment, respectively. Arrowheads indicate cell nuclei.

Previous studies have used adenoviral-enhanced yellow fluorescent protein-tagged-human CAR (Ad-EYFP-hCAR) as a tool to visualize nuclear translocation of hCAR from cytoplasm in primary human hepatocytes upon exposure to phenobarbital (Li et al., 2009). These studies demonstrated the translocation phenomenon in primary human hepatocytes and intact liver, but not in immortalized cells such as HepG2, where spontaneous accumulation of hCAR in the nucleus leads to constitutive CAR activation in the absence of a chemical inducer. The same Ad-EYFP-hCAR fusion protein model system was used to study CAR nuclear translocation in HepatoCells. A 3-day culture of HepatoCells was transduced with Ad-EYFP-hCAR for 24 hours. Infected HepatoCells were then treated with 1 mM phenobarbital for 12 hours. Prior to treatment, EYFP-hCAR expression in both primary hepatocytes and HepatoCells is mostly excluded from the cell nucleus, as shown by strong fluorescent signal in the cytoplasm (Fig. 3, A and C); similar to primary human hepatocytes, fluorescent EYFP-hCAR relocates to the cell nuclei following treatment (Fig. 3, B and D). This observation suggests that HepatoCells maintain primary human hepatocyte-like phenobarbital-responsive hCAR nuclear translocation.

#### Concentration-Dependent CYP3A4 Induction Response.

After initial evaluation of HepatoCells for induction response to single concentration of positive control inducers, HepatoCells were subsequently tested for response to a group of known clinical inducers (or noninducers) at different concentrations. Eighteen compounds were chosen, including strong, moderate/weak, and noninducers, based on their potency to reduce AUC of victim drugs in clinical studies (Zhang et.al. 2014). Three lots of HepatoCells (lots 2B, 3A, and 3B) were treated for 3 consecutive days with the test compounds at eight concentrations for each compound. Both CYP3A4 enzymatic activity and mRNA expression were measured. Concentration-dependent CYP3A4 induction response curves were generated using fold increase data from both enzymatic activity and mRNA expression. It is well known that intracellular drug concentration may be different from nominal drug concentration during the 24-hour incubation period due to various reasons such as metabolism, nonspecific binding to culture surface, degradation, etc.; therefore, EMA guidance recommends estimating actual drug exposure by measuring the drug concentration in culture medium over time. However, in a recent article, Zhang et al. (2014) reported that using time-weighted average concentrations to derive induction parameters did not offer any improvement in prediction accuracy; therefore, in our current study, we use nominal concentrations to derive EC_50_ and E_max_.

**Fig. 4.**
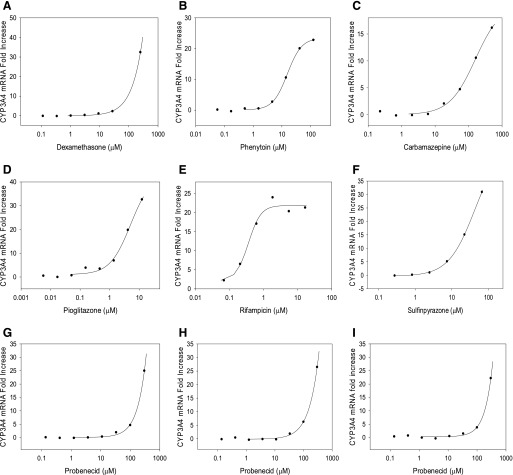
Examples of concentration-dependent induction response curves used to determine E_max_ and EC_50_ using fold increase data estimated with CYP3A4 mRNA expression level. Graphs (A–F) are examples of concentration-dependent induction response curves for six compounds in lot 2B HepatoCells. Graphs (G–I) are examples of probenecid concentration-dependent induction response curves in lots 2B, 3A, and 3B HepatoCells.

As expected, all four in vitro noninducers, flumazenil, primaquine, methotrexate, and digoxin, showed no induction response in either CYP3A4 enzyme activity or mRNA expression (Supplemental Tables 1 and 2). All compounds that are categorized as clinical inducers showed concentration-dependent response with greater than twofold increase in both enzyme activity and mRNA expression over solvent vehicle control—demonstrating positive induction response, according to EMA guidance (http://www.ema.europa.eu/docs/en_GB/document_library/Scientific_guideline/2012/07/WC500129606.pdf). Figure 4, A–F, shows examples of fold induction changes over a range of concentrations for six model compounds based on mRNA data. All curves were fitted using sigmoidal Hill 4 parameter function of SigmaPlot with R^2^ all greater than 0.9 (Table 2). Slope factors were reported in Supplemental Table 3. It is noted that the slope factors are in the range of 0.4–4.2, which is also seen in primary hepatocytes. Such large range of slope factors could potentially impact prediction outcome; however, very few discussions were reported on the role of the slope factors, and no validated method is available to incorporate slope factors into induction prediction; therefore, we chose to follow the conventional method used by the industry as in published reports to not consider slope factors when modeling the prediction for a like-for-like comparison between the alternative model HepatoCells and the gold standard primary hepatocytes.

**TABLE 2:** E_max_ and EC_50_ determination using concentration-dependent induction response curves based on CYP3A4 mRNA induction fold increase data Fold increase = fold induction, 1.

	Test Concentration	Lot 2B			Lot 3A			Lot 3B		
		R^2^	E_max_	EC_50_	R^2^	E_max_	EC_50_	R^2^	E_max_	EC_50_
Test Compounds	*μ*M		Fold	*μ*M		Fold	*μ*M		Fold	*μ*M
Rifampicin	0.01– 50	0.98	22	0.36	0.96	28	0.27	0.98	26	0.52
Phenytoin	0.6–120	1.00	23	15	1.00	38	16	0.99	35	23
Carbamazepine	0.23–500	1.00	16	108	1.00	10	65	0.98	14	53
Phenobarbital	0.91–2000	1.00	110	558	1.00	135	728	1.00	73	775
Terbinafine	0.05–100	1.00	20	3.6	1.00	24	2.2	0.99	21	2.7
Sulfinpyrazone	0.09–200	1.00	31	23	0.98	53	28	0.98	38	32
Probenecid	0.13–300	1.00	25	187	1.00	26	176	1.00	22	195
Pioglitazone	0.006–12.5	0.99	33	3.3	0.99	31	2.3	0.98	15	2.0
Dexamethasone	0.11–250	1.00	32	139	1.00	53	117	1.00	29	134
Rosiglitazone	0.05–100	0.99	49	3.8	1.00	85	4.3	1.00	95	4
Omeprazole	0.05–100	1.00	24	13	1.00	45	23	1.00	27	13
Clotrimazole	0.005–10	1.00	16	0.58	0.99	18	0.35	1.00	17	0.33
Nifedipine	0.05–100	1.00	28	5.6	0.99	40	6.6	0.99	27	3.5
Quinidine	0.11–250	NA	1.0	NA	NA	2.0	NA	NI	1.12	NI
Flumazenil	0.023–50	NI	NI	NI	NI	NI	NI	NI	NI	NI
Primaquine	0.05–40	NI	NI	NI	NI	NI	NI	NI	NI	NI
Methotrexate	0.009–20	NI	NI	NI	NI	NI	NI	NI	NI	NI
Digoxin	0.0002–0.2	NI	NI	NI	NI	NI	NI	NI	NI	NI

NA, not able to conduct curve fitting to obtain EC_50_ and E_max_ due to no concentration-dependent response curve was obtained (only observed maximum fold induction is reported); NI, no induction observed at tested concentration.

Curves generated using enzyme activity data showed similar good fitting (data not shown). Three different lots of HepatoCells showed comparable concentration-dependent induction response to model compounds, for example in Fig. 4, G–I, lots 2B, 3A, and 3B responded to probenecid with E_max_ of 25-, 26-, and 22-fold, respectively (Table 2), and R^2^ values were all 1.00, suggesting consistent performance of HepatoCells.

EC_50_ and E_max_ were determined for compounds that exhibited a typical sigmoid-shaped dose-response characteristic of nuclear-dependent pathway (Table 2). For compounds that do not show a plateau, E_max_ is estimated as the observed maximum induction response to avoid extrapolating too further away from experimental values, and EC_50_ is estimated accordingly using the fitted curve. Among the 18 compounds tested, no EC_50_ and E_max_ data were generated for the four in vitro noninducers, flumazenil, primaquine, methotrexate, and digoxin (as no induction response was observed), or for the clinical noninducer quinidine (Leizorovicz et al., 1984; Mihaly et al., 1987) as it did not cause a concentration-dependent increase in either CYP3A4 enzyme activity or mRNA expression from any of the three batches of HepatoCells, even though at a couple of concentrations a one- to twofold increase was observed (Supplemental Induction Data). Another clinical noninducer clotrimazole (Shord et al., 2010) only induced an increase in CYP3A4 mRNA expression (therefore, EC_50_ and E_max_ were determined), consistent with reported in vitro studies using primary human hepatocytes and hepatocyte cell line (Raucy, 2003; Ripp et al., 2006), but did not increase CYP3A4 enzyme activity. A third clinical noninducer, nifedipine, showed positive induction response in both enzyme activity and mRNA level as indicated by greater than 1.4-fold induction and a dose-dependent pattern. Therefore, a concentration-dependent response curve was generated and induction parameters were calculated for this compound.

#### Generation of RIS Calibration Curves and Prediction of Clinical Inducers.

**Fig. 5.**
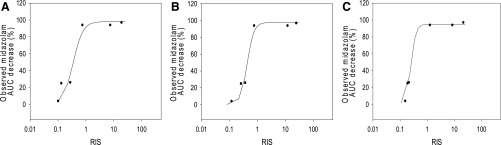
Calibration curves of in vivo midazolam AUC change (%) as a function of RIS, using data from CYP3A4 mRNA induction response in three different lots of HepatoCells, lot 2B (A), lot 3A (B), and lot 3B (C). In vivo midazolam AUC change (%) was obtained from a previous study (Zhang et al., 2014).

Induction parameters RIS were calculated using the formula described in *Materials and Methods*. In this study, unbound plasma C_max_ was used according to the recommendation in EMA DDI guidance (http://www.ema.europa.eu/docs/en_GB/document_library/Scientific_guideline/2012/07/WC500129606.pdf). Calibration curves were then established for each of the three lots of HepatoCells by plotting the induction parameters RIS against observed decrease in midazolam AUC (Fig. 5). All three curves showed good correlation between RIS and the observed midazolam AUC change. Specifically, R^2^ values of 0.95, 0.97, and 0.99 were calculated for lots 2B, 3A, and 3B, respectively.

Similar to reported RIS data using primary hepatocytes (Zhang et al., 2014), HepatoCells demonstrated high RIS values for strong clinical inducers. For example, RIS values were 0.8–25 for strong inducers, including rifampicin, phenytoin, and carbamazepine, when calculated based on CYP3A4 mRNA level, and were 0.7–24 when calculated based on enzyme activity (Table 3). For the clinical noninducers omeprazole, nifedipine, and dexamethasone, RIS values were low, in the range of 0.0004–0.09 when measured using CYP3A4 enzyme activity and 0.0012–0.16 when measured using CYP3A4 mRNA. As expected, the three lots of HepatoCells showed small variation in RIS, with an average CV of 23% when using enzyme activity, and 24% when using mRNA.

**TABLE 3:** RIS values calculated using induction response measured by CYP3A4 mRNA level and enzyme activity in HepatoCells

Compound	RIS Based on mRNA					RIS Based on Enzyme Activity				
	Lot 2B	Lot 3A	Lot 3B	Mean	%CV	Lot 2B	Lot 3A	Lot 3B	Mean	%CV
Rifampicin	19.1	24.8	21.5	21.8	13%	23.8	23.0	18.3	21.7	14%
Phenytoin	7.5	12.1	8.5	9.4	26%	1.8	2.3	2.4	2.2	13%
Carbamazepine	0.76	0.77	1.3	0.9	31%	0.67	0.8	0.9	0.8	17%
Phenobarbital	5.2	5.0	2.5	4.3	35%	2.9	4.2	4.0	3.7	18%
Terbinafine	0.13	0.26	0.19	0.19	34%	0.15	0.23	0.17	0.2	21%
Pioglitazone	0.28	0.37	0.21	0.29	28%	0.12	0.20	0.28	0.2	42%
Sulfinpyrazone	1.2	1.8	1.1	1.4	25%	1.5	1.4	1.2	1.3	10%
Probenecid	2.8	3.2	2.4	2.8	13%	1.5	1.0	0.8	1.1	33%
Dexamethasone	0.0013	0.0024	0.0012	0.0016	43%	0.00054	0.00041	0.00043	0.0005	15%
Nifedipine	0.10	0.12	0.16	0.13	22%	0.09	0.09	0.04	0.0697	39%
Rosiglitazone	0.043	0.065	0.070	0.059	25%	0.019	0.028	0.024	0.0237	18%
Omeprazole	0.069	0.073	0.079	0.074	7%	0.015	0.009	0.007	0.0103	39%
Clotrimazole	0.0028	0.0051	0.0052	0.0044	7%	NA	NA	NA		
Mean %CV					24%					23%

According to FDA definition of clinical DDI (Ratio of the areas under the concentration–time curve (AUCR) = 0.8–1.25) and following industry standard practice (Einolf et al., 2014; Zhang et al., 2014; Almond et al., 2016; Fahmi et al., 2016; Vermet et al., 2016), we calculate RIS cutoff value for a positive inducer when the values leading to a 20% decrease in predicted victim drug AUC change. RIS cutoff at 20% AUC change was calculated for all three lots of HepatoCells. The values are similar whether enzyme activity or mRNA expression level was used. Specifically, RIS cutoff values are 0.17 and 0.23 using the induction parameter generated with enzyme activity and mRNA expression, respectively (Table 4). This was also reported for primary hepatocytes, in which the mean cutoff values were determined to be 0.013 and 0.016 based on enzyme activity and mRNA expression, respectively (Zhang et al., 2014).

**TABLE 4:** RIS cutoff value at 20% midazolam AUC change in HepatoCells and primary human hepatocytes Primary human hepatocyte data were obtained from a previous study (Zhang et al., 2014).

HepatoCells			Primary Human Hepatocytes		
Lot No.	RIS (Enzyme Activity)	RIS (mRNA)	Lot No.	RIS (Enzyme Activity)	RIS (mRNA)
Lot 2B	0.12	0.20	Lot 295	0.017	0.017
Lot 3A	0.19	0.30	Lot 312	0.013	0.019
Lot 3B	0.20	0.19	Lot 318	0.0078	0.011
Mean	0.17	0.23	Mean	0.013	0.016
%CV	25%	26%	%CV	37%	27%

Table 5 shows that the predicted AUC changes using HepatoCells were similar to the observed AUC changes (Zhang et al., 2014) with a few exceptions. For strong inducers, HepatoCells predicted 95–98% AUC change for rifampicin, which caused 97% midazolam AUC change in clinical DDI studies. HepatoCells predicted 95–98% midazolam AUC change for phenytoin, which caused 94% midazolam AUC change in a clinical DDI study. For certain moderate and weak inducers, HepatoCells also showed good prediction. For example, pioglitazone is a clinical weak inducer causing a 26% midazolam AUC change in a clinical study; HepatoCells predicted a 29–36% AUC change using mRNA data, and predicted a 17–34% AUC change using enzyme activity data, correctly categorizing it as a weak inducer. For dexamethasone, omeprazole, and nifedipine, HepatoCells predicted zero, 0.1–1.7%, and 0.9–8% AUC change, respectively, correctly categorizing the three compounds as noninducers. For these compounds, prediction using HepatoCells was similarly accurate as primary hepatocytes (Zhang et al., 2014), with both models correctly categorizing these compounds. An exception is terbinafine, which is a clinical weak inducer causing a 25% midazolam AUC change in a clinical DDI study; HepatoCells predicted 15–29% midazolam AUC change for terbinafine using enzyme activity data and predicted 8–19% AUC change when using mRNA data, suggesting a moderate underestimation.

**TABLE 5:** Predicted AUC change using RIS calibration curve based on CYP3A4 mRNA and enzyme activity of HepatoCells Observed AUC change was obtained from a previous study (Zhang et al., 2014).

Compounds	Observed % AUC Change	Predicted % AUC Change Based on mRNA			Predicted % AUC Change Based on Activity		
		Lot 2B	Lot 3A	Lot 3B	Lot 2B	Lot 3A	Lot 3B
Rifampicin*	97	98	98	95	96	96	99
Phenytoin*	94	98	98	95	96	96	97
Carbamazepine*	94	86	87	95	92	92	87
Phenobarbital	61	98	98	95	96	96	98
Pioglitazone*	26	36	35	29	17	23	34
Terbinafine*	25	8.1	13	19	29	29	15
Sulfinpyrazone	22	94	97	95	96	95	92
Probenecid	20	98	98	95	96	94	83
Dexamethasone	19	0.00	0.00	0.00	0.0	0.0	0.0
Nifedipine*	4	4.4	0.89	8.3	8.9	2.1	0.63
Omeprazole	−25	1.7	0.14	0.35	0.11	0.002	0.017
Rosiglitazone	12	0.53	0.10	0.20	0.21	0.077	0.22
Clotrimazole	9.7	0.00	0.00	0.00	0	0	0

* Denotes that compounds were used to generate RIS calibration curves.

There are a few compounds that were overestimated. For example, phenobarbital is a moderate inducer causing 61% in vivo AUC change, whereas HepatoCells predicted 95–98% AUC change, potentially categorizing it as a strong inducer. Overestimation was also observed with the weak inducer sulfinpyrazone and probenecid using HepatoCells (Table 5).

To further evaluate prediction accuracy using HepatoCells as a model, we calculated accuracy and bias using the two parameters described previously (Einolf et al., 2014), RMSE and GMFE. According to the definition, greater accuracy is represented by lower RMSE, and the lowest GMFE value would represent the lowest prediction bias. Overall, no significant difference in prediction accuracy and bias was observed whether enzyme activity or mRNA level was used for induction response (Table 6). However, when using midazolam as the victim drug, prediction accuracy is significantly better and bias significantly lower than using nonmidazolam victim drugs, which is true for both HepatoCells and primary hepatocytes (Zhang et al., 2014). For example, when using nonmidazolam victim drugs, RMSE is 0.47–0.49 for HepatoCells based on induction response of mRNA level, which is 5–14 times higher than RMSE of 0.034–0.088 when using midazolam as the victim drug; similarly, GMFE is 44–587 when using nonmidazolam victim drugs, which is up to 489 times higher than GMFE of 1.2–1.5 when using midazolam as the victim drug. This analysis confirmed the above finding of overestimation of AUC change when nonmidazolam victim drugs were used.

**TABLE 6:** Prediction accuracy and bias in the prediction of clinical CYP3A4 inducers using HepatoCells

Substrate	Metrics	Endpoint	HepatoCells		
			Lot 2B	Lot 3A	Lot 3B
Midazolam and nonmidazolam	RMSE	Activity	0.34	0.33	0.31
		mRNA	0.34	0.35	0.33
	GMFE	Activity	7.9	12.3	8.4
		mRNA	6.5	13.0	20.1
Midazolam	RMSE	Activity	0.046	0.025	0.063
		mRNA	0.088	0.070	0.034
	GMFE	Activity	1.3	1.2	1.6
		mRNA	1.3	1.5	1.2
Nonmidazolam	RMSE	Activity	0.48	0.47	0.43
		mRNA	0.48	0.49	0.47
	GMFE	Activity	71	207	62
		mRNA	44	169	587

**Fig. 6.**
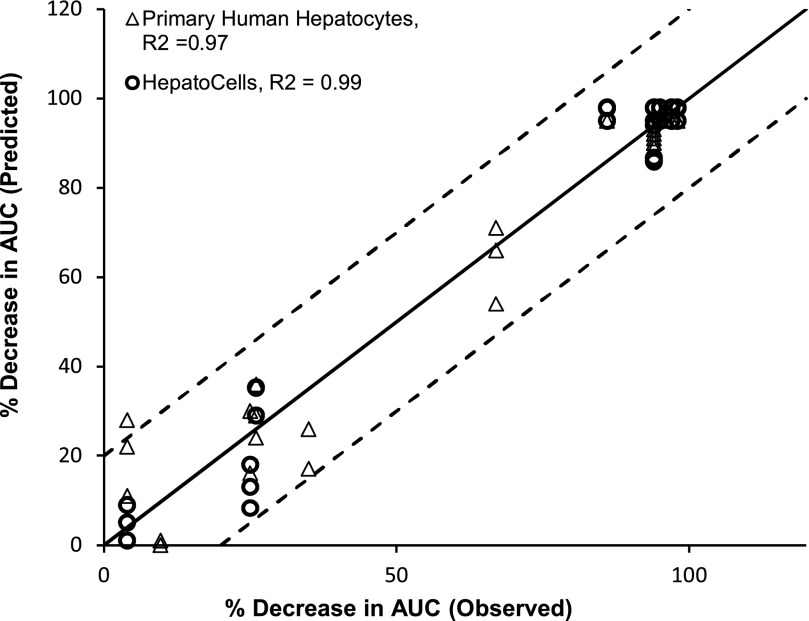
Correlation analysis of observed midazolam AUC change (%) and predicted AUC change (%) using HepatoCells and primary hepatocytes. Observed midazolam AUC change (%) and primary human hepatocyte data were obtained from a previous study (Zhang et al., 2014).

The predicted AUC change was plotted against observed AUC change. Figure 6 showed that both Corning HepatoCells and primary human hepatocytes correlated well with the line of unity with R^2^ greater than 0.9, and both fell within 20% of observed value for most of the test compounds, again suggesting similarly good prediction accuracy.

## Discussion

The present study was designed to fully characterize HepatoCells for its applicability as an in vitro tool for screening potential CYP inducers. We have shown that HepatoCells maintain primary human hepatocyte-like morphology, and retain primary hepatocyte-like capability to respond to positive control inducers of all three important CYPs (CYP3A4, 1A2, and 2B6). It is well known that ligand-activated nuclear receptors play a central role in regulating transcriptional expression of numerous drug-metabolizing enzymes and transporter proteins; for example, PXR, CAR, and aryl hydrocarbon receptor are the major xenobiotic receptors responsible for regulation of CYP3A4, 2B6, and 1A2, respectively. However, accumulating evidence has shown cross-talk between nuclear receptors, for example, CAR signaling pathway also contributes to the regulation of *CYP3A4* gene expression and enzyme activity, and PXR activation contributes to the induction of CYP2B6 as well, although at a lesser degree than CYP3A4 induction (Faucette et al., 2007; Lim and Huang, 2008). This suggests that using a cell model lacking the CAR regulation pathway poses the risk of missing potential clinical inducers. The fact that HepatoCells demonstrate phenobarbital-responsive nuclear translocation of CAR, a feature characteristic of primary human hepatocytes and lost in many hepatocyte cell lines, makes HepatoCells an attractive model for screening in vivo inducers especially when induction pathways other than PXR activation are involved.

It is noticed that compounds exhibit various patterns of concentration-dependent curves (Fig. 4, A–F). Some compounds like rifampicin form a plateau, showing clear E_max_ and are easy to derive EC_50_ (Fig. 4E). Some compounds do not reach a plateau at tested concentrations. The possible reasons include cytotoxicity, insolubility, and enzyme inhibition at high concentrations. For example, pioglitazone was insoluble at 33 *μ*M and 100 *μ*M, whereas at concentrations of 0.006–12.5 *μ*M, pioglitazone caused linear increase in induction response without reaching a plateau, resulting in a half S-shape (Fig. 4D). Note the curve-fitting function (sigmoidal Hill 4 parameter function) measures slopes of the fitted curves, which, depending on which part of the same data set is used for curve fitting, could vary significantly. Ideally, the curve-fitting slopes should be considered in a prediction model; however, the practical use has not been adopted by the pharmaceutical industry (Chu et al., 2009). There are very few discussions on how the curve-fitting slopes may be used in prediction models, possibly because the current practice of not considering this factor has generated sufficient prediction accuracy.

HepatoCells closely model the behavior of primary human hepatocytes during induction treatment. Because previous studies comparing different models from simple to complex suggested that a calibration-based approach provides sufficient prediction (Einolf et al., 2014), we chose the RIS model for the present study. Using the calibration-based approach, HepatoCells demonstrate prediction capability very close to primary human hepatocytes. For example, all three strong inducers, rifampicin, carbamazepine, and phenytoin, demonstrate predicted percentage of AUC change very similar to the observed values using both cell types. Previous study has shown that clotrimazole inhibits CYP3A4 activity by tight binding with a very small Ki of 0.25 nM (Gibbs et al., 1999). Because HepatoCells were treated with clotrimazole at concentrations between 10 nM and 10 *μ*M in the present study, it is likely that clotrimazole acted as a potent CYP3A4 inhibitor masking the induction in enzyme activity; hence, no concentration-dependent response was observed. Similarly, the positive induction response in CYP3A4 mRNA level caused by clotrimazole treatment of HepatoCells was also observed in primary human hepatocytes; however, unlike HepatoCells, two of three lots of primary human hepatocytes tested also demonstrated positive induction response in CYP3A4 enzyme activity, albeit to a moderate degree (E_max_ = 3.1- to 3.3-fold) (Zhang et al., 2014). Nifedipine is a clinical noninducer; however, in the present study, it caused induction in both enzyme activity and mRNA transcript expression, as indicated by E_max_ of greater than 20-fold in HepatoCells. This result is consistent with the findings in primary hepatocytes where mRNA transcript levels increased by three- to eightfold and enzyme activity increased by 1.4- to threefold (Zhang et al., 2014).

A few test compounds were predicted to cause higher AUC changes than clinical DDI studies, including the clinical noninducer omeprazole, the moderate inducer phenobarbital, and weak inducers probenecid and sulfinpyrazone. All these examples of overestimation share the common feature that nonmidazolam drugs were used as substrates in the clinical DDI studies, for example, carbamazepine as a substrate to assess probenecid effect (Kim et al., 2005), R-warfarin as a substrate to assess sulfinpyrazone effect (O’Reilly, 1982), and nifedipine as a substrate to assess phenobarbital effect (Schellens et al., 1989) and omeprazole effect (Soons et al., 1992). This same finding was previously reported in primary hepatocytes (Zhang et al., 2014). When prediction accuracy was analyzed using the metrics GMFE and RMSE, it also clearly demonstrated that a calibration curve based on midazolam as the victim substrate can accurately predict induction using midazolam as substrate, but not for induction using nonmidazolam substrate. Combined together, it is not suggested to generate a calibration curve using in vitro data from one substrate and apply such calibration curve for prediction of induction involving a different substrate.

The overestimation of probenecid induction is worth a closer look. Probenecid caused a marked induction response with E_max_ at 5.9- to 11.9-fold for enzyme activity and more than 20-fold for mRNA expression. This seems to be contradictory to a previous report by Luo et al. (2002), where probenecid was used as a negative control and no activation of PXR or induction of CYP3A4 transcript or enzyme activity was observed at probenecid concentration up to 50 *μ*M for PXR reporter gene assay or up to 20 *μ*M for CYP3A4 enzyme activity assay. In our test, HepatoCells did not exhibit significant induction response (>twofold) when probenecid was at 0.1–11 *μ*M, which is a similar range when it is used as a negative control (Luo et al., 2002); however, when probenecid concentration increased to higher than 33 *μ*M, probenecid started to demonstrate strong induction in our test. It is worth noting that probenecid has high unbound C_max_ (28 *μ*M); therefore, it is important to use a concentration range large enough to cover this value to assess induction potential.

Other renewable in vitro models such as HepaRG (a hepatoma-derived cell line) and Fa2N4 (an immortalized human hepatocyte cell line) have been tested as substitutes for primary human hepatocytes for modeling CYP3A4 induction DDI (Ripp et al., 2006; Kanebratt and Andersson 2008). However, limitations of HepaRG include mixed cell populations and the required use of DMSO for differentiation and maintaining drug metabolic activities. A limitation of the Fa2N4 cell line is the lack of a relevant CAR signaling pathway. Moreover, induction studies revealed that Fa2N4 cells have greater than 10 times higher EC_50_ value for rifampicin compared with primary hepatocytes, which was considered to be due to low expression of the uptake transporter OATP1B1/1B3 (Hariparsad et al., 2008). In contrast, HepatoCells was shown to retain primary hepatocyte-like phenobarbital-responsive CAR nuclear translocation. HepatoCells have similar EC_50_ values as primary hepatocytes (Zhang et al., 2014), with most values within a two- to threefold difference of each other. For example, HepatoCells showed EC_50_ values for rifampicin of 0.27–0.52 (Table 2), whereas primary hepatocytes showed EC_50_ values of 0.12–1.4 (Zhang et al., 2014). In addition, HepatoCells were tested for drug uptake activity using substrates for OATP1B1/1B3 and OCT1, demonstrating kinetic values (K_m_) similar to both native (primary human hepatocytes) and recombinant systems (data to be presented in a separate publication), suggesting that HepatoCells actively express functional uptake transporters.

Currently, primary human hepatocytes are the preferred model for in vitro testing of drug metabolism and toxicity profiles. However, its use is limited due to large lot-to-lot limitations. Compared with primary human hepatocytes, HepatoCells demonstrate much better performance consistency, as indicated by 5–8 times lower lot-to-lot variations in fold induction values of all three enzymes (Table 1); much smaller variation in RIS values, that is, 7–43% CV for HepatoCells RIS data based on mRNA expression (Table 3) versus 9–115% CV for primary hepatocytes (Zhang et al., 2014); and smaller variation in 20% AUC cutoff (Table 4). Overall, this suggests that, although primary hepatocytes are the preferred model for the definitive study of DDI required for new drug application submission, HepatoCells is a better tool for early stage screening due to better reproducibility. However, it has to be noted that HepatoCells are derived from a young donor, which may limit its use in some in vitro ADME studies. It is a general perception that primary hepatocytes from younger donors have higher chance of success of immortalization than cells from adult donors; however, we have recently achieved successful immortalization of primary hepatocytes from several adult donors; thus, cell lines generated with a broad range of donor demographic profiles could provide more options as primary hepatocyte alternative.

In conclusion, as a renewable hepatocyte model that closely mimics the behavior of primary hepatocytes, but with much higher reproducibility and reliable supply, HepatoCells is considered a useful in vitro tool for early stage screening.

## Abbreviations

AUC: area under curve
CAR: constitutive androstane receptor
CDFDA: carboxy-dichlorofluorescein diacetate
CYP: cytochrome P450
DDI: drug-drug interaction
DMSO: dimethylsulfoxide
E_max_: maximum response
EMA: European Medicines Agency
FDA: Food and Drug Administration
GMFE: geometric mean fold error
hCAR: human CAR
MRP2: multidrug-resistant protein 2
PXR: pregnane X receptor
R^2^: correlation coefficient
RIS: relative induction score
RMSE: root mean square error
